# A prognostic nomogram for the cancer-specific survival rate of choroidal melanoma using the Surveillance, Epidemiology, and End Results database

**DOI:** 10.3389/fmed.2024.1392336

**Published:** 2024-05-16

**Authors:** Xianfen Cao, Jing Zeng, Yulun Ou, Jian Chen, Jun Lyu, Qing Zhou

**Affiliations:** ^1^Department of Ophthalmology, The First Affiliated Hospital of Jinan University, Guangzhou, China; ^2^Department of Ophthalmology, The First People’s Hospital of Chenzhou, Chenzhou, China; ^3^Ophthalmic Center, The Second Affiliated Hospital, Guangzhou Medical University, Guangzhou, China; ^4^Department of Clinical Research, The First Affiliated Hospital of Jinan University, Guangzhou, China

**Keywords:** choroidal melanoma, nomogram, cancer-specific survival, prognosis, SEER

## Abstract

**Objective:**

This study was conducted to develop a comprehensive nomogram for individuals with choroidal melanoma (CM) to determine their cancer-specific survival (CSS).

**Methods:**

Data of individuals with CM, diagnosed between 2004 and 2015, were accessed at the Surveillance, Epidemiology, and End Results (SEER) database. The selected individuals were randomly categorized into a training and validation cohort. Multivariate Cox regression analysis was applied to screen the relevant variables. Followed by the development of a nomogram based on independent variables. Ultimately, the net reclassification index (NRI), concordance index (C-index), calibration charts, integrated discrimination improvement (IDI), receiver operating characteristic curves (ROC), area under the curve (AUC), and decision-curve analysis (DCA), were utilized to evaluate the discrimination, accuracy, and effectiveness of the model.

**Results:**

This study enrolled 3,782 patients. Seven independent factors linked to prognosis were screened via multivariate Cox regression analysis, encompassing age at diagnosis; race; AJCC (American Joint Committee on Cancer) stage; histologic type; and therapy method of radiotherapy, surgery, and chemotherapy. The respective C-indexes of the training and validation cohorts were 0.709 and 0.726, indicative of the excellent accuracy of the nomogram. Furthermore, the AUCs of the training and validation cohorts across 3, 5, and 8 years were 0.767, 0.744, and 0.722 as well as 0.772, 0.770, and 0.753, respectively. Evident of the superiority of the established nomogram over the AJCC staging, both the NRI and IDI values exhibited improvement. The favorable clinical impact and good performance of the nomogram were evident via decision curve analyses (DCAs) and calibration plots, respectively.

**Conclusion:**

This research dealt with establishing and validating a nomogram as a prognostic tool for assessing the prognosis of adult patients with CM utilizing the SEER database. A comprehensive assessment of the nomogram via diverse variables demonstrated its accuracy in predicting the CSS probabilities of CM patients across 3, 5, and 8 years in clinical settings. Notably, its performance surpassed that of the AJCC staging system.

## Introduction

Uveal melanoma is a highly prevalent form of primary intraocular malignancy in adults, exhibiting an annual incidence of 5–10 cases per million individuals globally (1–3). Uveal melanomas can occur anywhere in the uveal tract, with the choroid accounting for 90% of cases, the ciliary body for 6%, and the iris for 4% (1). In most instances, the diagnosis of uveal melanoma and the determination of treatment planning can be achieved based on comprehensive exam of both the anterior and posterior segments of the eye, such as ultrasonography, fundus photography, optical coherence tomography (OCT) and autofluorescence. These multimodal imaging, when administered in a timely manner, can significantly contribute to an improvement in survival rates thanks to early detection (4, 5). Treatment started with enucleation and has progressed to eye-sparing treatments (i.e., radiotherapy, local tumour resection and phototherapy), allowing patients to preserve remarkable long-term vision (6). However, approximately 50% of patients suffering from uveal melanoma develop metastases, which develop most in the liver, resulting in a significantly poor prognosis; the median overall survival is approximately 1 year (7). Despite advances in targeted therapy, immune therapy and other new therapeutic perspectives of uveal melanoma, the survival rates have not improved significantly (6, 8).

Presently, several studies have adopted a comprehensive approach, considering all locations of uveal melanoma within one cohort to analyze the prognostic factors (9–11). Nevertheless, prior research has put forth new evidence potentially correlating the survival outcomes with the specific location of the primary tumor. Iris melanomas are typically discovered early, resulting in a favorable outcome; while ciliary body melanomas are associated with an adverse prognosis since they are difficult to detect (8, 12–14). Liang et al. (15) conducted a study revealing that notably better survival rates were associated with choroidal melanoma (CM) in comparison to individuals with iris/ciliary body melanoma. Considering the substantial impact of the tumor location on the prognosis of uveal melanoma, relying on a consolidated prediction model for assessing overall survival may result in unique factors being overlooked. This may inevitably lead to the misestimation of survival in some CM patients. Consequently, it is essential to conduct separate analyses for patients with CM to account for the unique characteristics associated with this specific location and ensure a more precise evaluation of their survival prospects.

A nomogram serves as a reliable tool capable of visually assessing risks by incorporating important pathological and clinical variables linked to oncologic outcomes (16). Notably, nomograms have shown greater precision in predicting outcomes for diverse malignancies, such as cutaneous verrucous carcinoma, thyroid carcinoma, and gastric cancer in comparison to the traditional American Joint Committee on Cancer (AJCC) staging system (17–19). This underscores their valuable contribution to the advancement of personalized oncology strategies. Per the extensive literature assessed, no prior study appears to have established a nomogram for CM patients. Therefore, herein, using data acquired from the Surveillance, Epidemiology, and End Results database (SEER), we aimed to develop a predictive nomogram for adult patients with CM. Additionally, a multidimensional validation was carried out to thoroughly examine the good predictive efficacy of the model, which will provide objective and scientifically grounded guidance for clinical decision-making.

## Methods

### Data source and patient selection

The time period of 2004 to 2015 was assessed for CM-related cases across 17 distinct registries of the SEER program. This population-based database encompasses 17 cancer registries across the US, overseen by the National Cancer Institute. Covering nearly 35% of the population, it provides a representative reflection of the demographics of the country (16). For data extraction, the SEER*stat software 8.4.3 was utilized with the case listing option. The relevant individuals were selected utilizing the International Classification of Disease for Oncology, third edition (ICOD-3) codes: 8,720–8,790 for malignant melanoma and C69.9 for choroid as the primary tumor site. Moreover, only patients who were diagnosed with CM between 2004 and 2015 were included. The exclusion criteria were as mentioned: (1) patients aged <18 years, (2) those who had not undergone any form of treatment, (3) those with missing or unknown AJCC and SEER stage, (4) those exhibiting a survival time <1 month, (5) those with missing or unknown SEER cause-specific death classification.

### Variable selection

This study collected information on variables like age at diagnosis, sex, race, marital status, laterality, histologic type, AJCC stage, SEER stage, radiotherapy, surgery, chemotherapy, SEER cause-specific death classification, and survival time (months). Cancer-specific survival (CSS) was designated as the outcome-predicting variable. AJCC staging was conducted following the guidelines of the 6th edition of the AJCC staging system (2004–2015). The technique of integrating histologic subtypes of patients with choroidal melanoma is based on the criterion of Liu et al. (9). Therefore, we have merged the NOS group and the other group (achromic melanoma, desmoplastic melanoma and nodular melanoma) into a unified group.

### Nomogram development and statistical analyses

The initial step in the process of establishing and validating the nomogram was the random allocation of the selected individuals into the training (70%) and validation (30%) cohorts. The optical cut-off point for age was calculated via X-tile, resulting in the categorization of patients into three groups: 18–50, 51–71, and >71 years. Multivariate Cox proportional hazards regression analyses, coupled with the stepwise selection method, were utilized to screen variables markedly influencing CM CSS. Additionally, during this analysis, hazard ratios (HR) and their associated 95% confidence intervals (CI) were concurrently recorded. Incorporating the screened prognostic factors, we established a nomogram for predicting survival across 3, 5, and 8 years in individuals with CM. The individual risk score was calculated utilizing the formula of the nomogram.

Net reclassification index (NRI) and integrated discrimination improvement index (IDI), relatively recent quantitative evaluation indicators, were employed to ascertain if the prognostic capacity of the model exhibited improvement in comparison to earlier models. This evaluation was carried out in a more thorough and multilevel manner. The concordance index (C-index), ROC curves, and the area under the ROC curve (AUC) were employed to examine the discriminatory capacity of the nomogram. Furthermore, calibration curves were employed to compare the correlation between the predicted and actual outcomes. Moreover, for evaluating the clinical utility, the net clinical benefit of the nomogram was comparatively assessed with that of the AJCC staging system via the decision curve analysis (DCA). DCA, a novel algorithm, examines the net benefit value of the model across diverse thresholds. The statistical analyses were carried out via the software package R (v 4.2.2), *p* < 0.05 were deemed to reflect statistical significance.

### Institutional Review Board approval

This study was strictly abide by the Declaration of Helsinki and exempted from Institutional Review Board oversight from the First Affiliated Hospital of Jinan University for the reason that patient information in the SEER program is de-identified and publicly available.

## Result

### Patient characteristics

This investigation enrolled 3,782 adults with CM who underwent random categorization into a training (*n* = 2,647) and validation (*n* = 1,135) cohort. Table 1 summarizes the demographic and clinical features of the selected individuals. Among the included patients, a majority were aged between 51–71 years (*n* = 2,023, 53.5%), were white (*n* = 3,667, 97%), and were married (*n* = 2,349, 62.7%), with the males slightly higher in number (52.6%) than the females (47.4%). Almost all patients experienced monocular onset (99.9%) of CM, and the right and left eyes were affected in 50.1 and 49.8% of patients, respectively. Most patients had AJCC stage I (35.5%) and stage II (44.6%), while, stage IV accounted for only 1.5% of the cases. Coincidentally, the SEER stage was predominated in location (93.1%), and the incidence of distant metastases was relatively low (1.5%). Histologic types encompassed spindle cell melanoma (8.1%), mixed epithelioid and spindle cell melanoma (7.7%), and epithelioid cell melanoma (2.7%). The NOS/other group accounted for 81.5%, with the majority being NOS (80.7%) and the remaining group comprised of other rare histological types (0.8%). Regarding treatment, the majority of patients received radiation treatment (79.4%), while 28.8 and 2.2% of patients underwent surgery and chemotherapy. Notably, no remarkable variation was observed in the percentage distribution of each indicator between the two cohorts.

**Table 1:** Clinicopathological information in choroidal melanoma.

	Overall (*N* = 3,782)		Training group (*N* = 2,647)		Validation group (*N* = 1,135)	
	Number	Percent	Number	Percent	Number	Percent
*Diagnosis of age*						
18–50	756	20%	509	19.2%	247	21.8%
51–71	2,023	53.5%	1,413	53.4%	610	53.7%
>71	1,003	26.5%	725	27.4%	278	24.5%
*Sex*						
Male	1,996	52.80%	1,374	51.9%	622	54.8%
Female	1,786	47.20%	1,273	48.1%	513	45.2%
*Race*						
White	3,667	97%	2,565	96.9%	1,102	97.1%
Black	25	0.7%	19	0.7%	6	0.5%
Other	90	2.4%	63	2.4%	27	2.4%
*Marital status*						
Married	2,349	62.1%	1,639	61.9%	710	62.5%
Single	490	13.%	337	12.7%	153	13.5%
DSW	669	17.7%	492	18.6%	177	15.6%
Other/unknown	274	7.2%	179	6.8%	95	8.4%
*Laterality*						
Left	1,893	50.1%	1,326	50.1%	567	49.95%
Right	1,883	49.8%	1,316	49.7%	567	49.95%
Bilateral/other	6	0.2%	5	0.2%	1	0.1%
*Histological type*						
Spindle	308	8.1%	208	7.9%	100	8.8%
Mixed	290	7.7%	200	7.6%	90	7.9%
Epithelioid	103	2.7%	68	2.5%	35	3.1%
NOS/other	3,081	81.5%	2,171	82%	910	80.2%
*AJCC stage*						
I	1,343	35.5%	936	35.4%	407	35.9%
II	1,687	44.6%	1,162	43.9%	525	46.2%
III	694	18.4%	509	19.2%	185	16.3%
IV	58	1.5%	40	1.5%	18	1.6%
*SEER stage*						
Location	3,521	93.1%	2,459	92.9%	1,062	93.6%
Regional	203	5.4%	148	5.6%	55	4.8%
Distant	58	1.5%	40	1.5%	18	1.6%
*Radiation*						
Yes	3,002	79.4%	2,093	79.1%	909	80.1%
No/unknown	780	20.6%	554	20.9%	226	19.9%
*Surgery*						
Yes	1,090	28.8%	775	29.3%	315	27.8%
No/unknown	2,692	71.2%	1,872	70.7%	820	72.2%
*Chemotherapy*						
Yes	84	2.2%	64	2.4%	20	1.8%
No/unknown	3,698	97.8%	2,583	97.6%	1,115	98.2%

### Variable selection

Table 2 summarizes the outcomes of the multivariate analyses of the training cohort. The stepwise analysis highlighted that age at diagnosis, race, AJCC staging, histological type, radiation, surgery, and chemotherapy functioned as independent variables. However, due to significant multicollinearity between AJCC and SEER staging, the SEER stage could not be established as an independent risk predictor of CM in adult patients. The final screening results encompassed various parameters and were as outlined: age, age 51–71 years (versus age 18–50 years: HR = 1.4795, 95% CI = 1.1815–1.8526, *p* < 0.001), age >71 years (versus age 18–50 years: HR = 2.045, 95% CI = 1.6022–2.6102, *p* < 0.001); race, black race (versus Caucasian people: HR = 0.356, 95% CI = 0.1142–1.1096, *p* = 0.075), other/unknown (versus Caucasian people: HR = 0.8993, 95% CI = 0.5287–1.5287, *p* = 0.695), Histological type, mixed (versus spindle: HR = 2.331, 95% CI = 1.6136–3.3672, *p* < 0.001), epithelioid (versus spindle: HR = 2.4938, 95% CI = 1.5632–3.9784, *p* < 0.001); NOS/other (versus spindle: HR = 2.0948, 95% CI = 1.5031–2.9193, *p* < 0.001); AJCC stage, AJCC stage II (versus AJCC stage I: HR = 1.5655, 95% CI = 1.2826–1.9107, *p* < 0.001), AJCC stage III (versus AJCC stage I: HR = 3.0945, 95% CI = 2.4930–3.8411, p < 0.001), AJCC stage IV (versus AJCC stage I: HR = 7.834, 95% CI = 5.1115–12.0066, *p* < 0.001); Treatments, no radiotherapy/unknown (versus radiotherapy: HR = 1.829, 95% CI = 1.3402–2.4961, *p* < 0.001), no surgery/unknown (versus surgery: HR = 0.7792, 95% CI = 0.5904–1.0283, *p* = 0.078), no chemotherapy/unknown (versus chemotherapy: HR = 0.5946, 95% CI = 0.3985–0.8871, *p* = 0.011).

**Table 2:** Selected variables by multivariable Cox regression analysis.

Multivariable analysis			
Variable	HR	95% CI	*p*-value
*Diagnosis of age*			
18–50	Reference		
51–71	1.4795	1.1815–1.8526	<0.001
>71	2.045	1.6022–2.6102	<0.001
*Race*			
White	Reference		
Black	0.356	0.1142–1.1096	0.075
Other/unknown	0.8993	0.5287–1.5298	0.695
*Histological type*			
Spindle	Reference		
Mixed	2.331	1.6136–3.3672	<0.001
Epithelioid	2.4938	1.5632–3.9784	<0.001
NOS/other	2.0948	1.5031–2.9193	<0.001
*AJCC stage*			
I	Reference		
II	1.5655	1.2826–1.9107	<0.001
III	3.0945	2.4930–3.8411	<0.001
IV	7.834	5.1115–12.0066	<0.001
*Radiation*			
Yes	Reference		
No/unknown	1.829	1.3402–2.4961	<0.001
*Surgery*			
Yes	Reference		
No/unknown	0.7792	0.5904–1.0283	0.078
*Chemotherapy*			
Yes	Reference		
No/unknown	0.5946	0.3985–0.8871	0.011

### Nomogram for CM CSS prognosis

**Figure 1:**
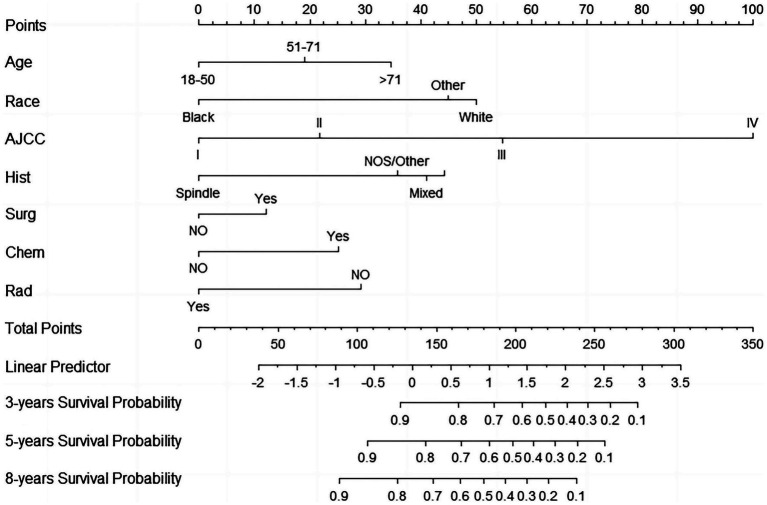
Prognostic nomogram for predicting 3-year, 5-year, and 8-year CSS of adult patients with CM.

A nomogram that incorporated the significant independent variables was developed in order to predict the survival outcomes of individuals with CM across 3, 5, and 8 years in the training cohort. Figure 1 illustrates the impact of various parameters on prognosis using the nomogram. The data highlights the substantial impact of AJCC staging, closely followed by race, histological types, age, radiotherapy, surgery, and chemotherapy. Every parameter in the nomogram received a certain score using a point system. By summing the scores for all the parameters, a vertical line was drawn to determine the cumulative score, indicating the CSS probabilities across 3, 5, and 8 years.

### Validation and calibration of the nomogram

**Figure 2:**
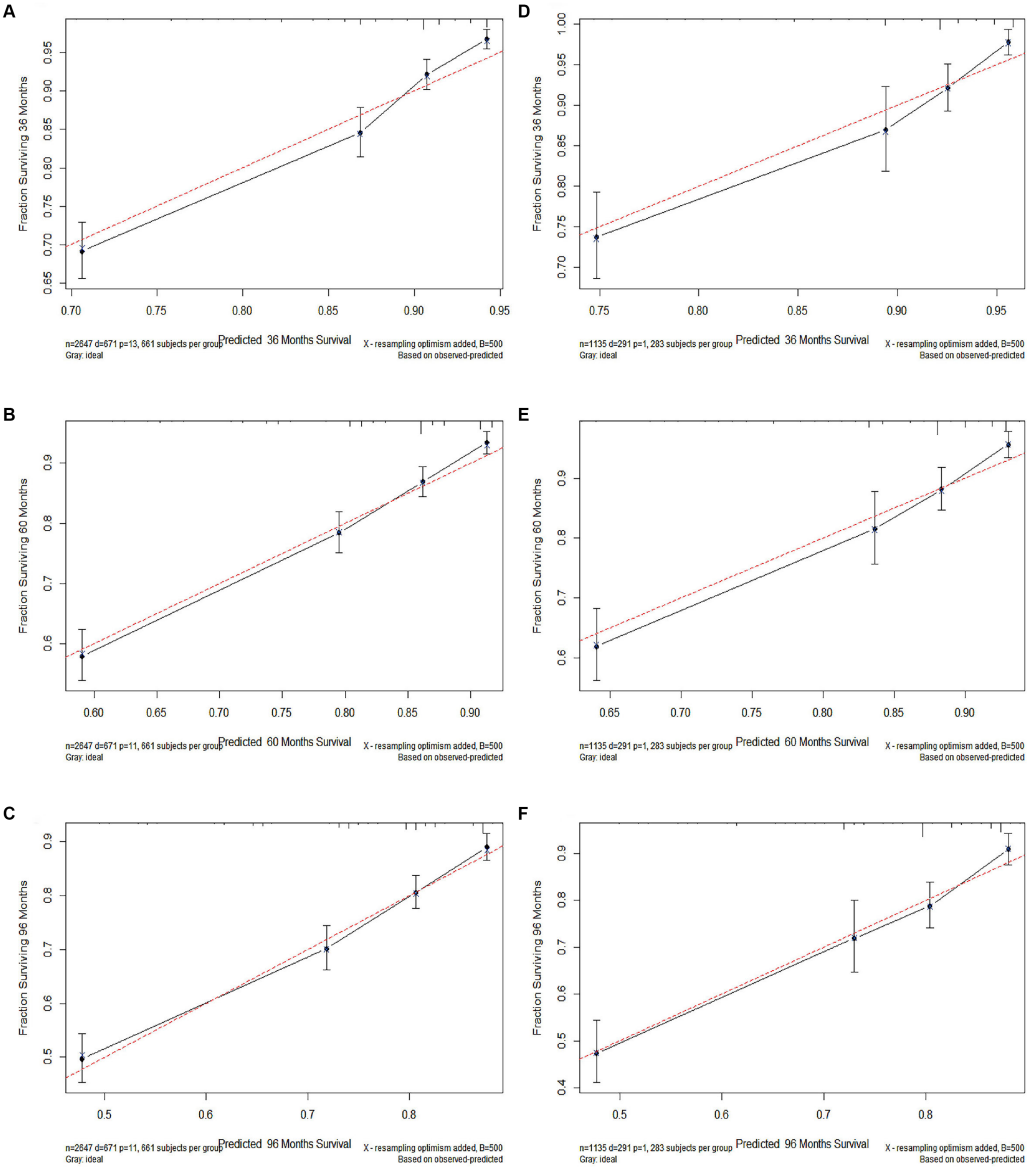
The calibration curves of 3-year **(A)**, 5-year **(B)**, and 8-year **(C)** cancer-specific survival (CSS) in the training cohort and 3-year **(D)**, 5-year **(E)**, and 8-year **(F)** CSS in the validation cohort.

**Figure 3:**
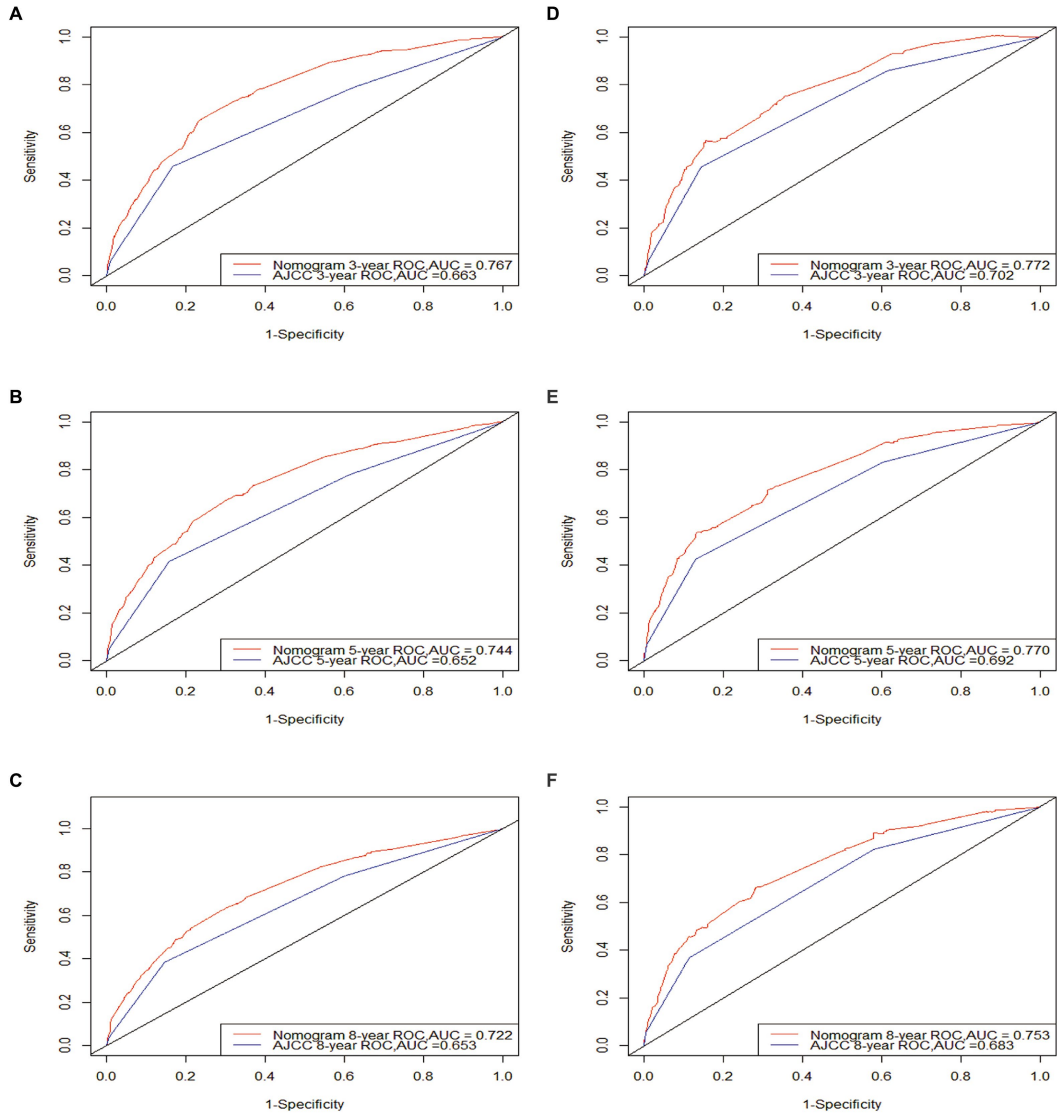
ROC curves. The area under the ROC curve (AUC) of the new nomogram compared to the traditional AJCC model. Both the training cohort **(A–C)** and validation cohort **(D–F)** demonstrate that the new nomogram is superior to AJCC in terms of predictive ability.

The calibration and discrimination of the nomogram were evaluated by undergoing testing through 500 bootstrap resamples. The C-indexes exhibited heightened values for the nomogram (0.709 and 0.726, respectively, for the training and validation cohorts) in comparison to the AJCC staging system (0.639 and 0.670, respectively). The calibration curve exhibited a strong alignment between the predicted and actual observed probabilities for adult patients with CM (Figure 2). The AUCs of the training and validation cohort at 3, 5, and 8 years were recorded to be 0.767, 0.744, and 0.722 as well as 0.772, 0.770, and 0.753, respectively. The AUC of the nomogram was observed to be >0.7 for CSS prediction over 3, 5, and 8 years in the two cohorts (Figure 3), signifying its effective discrimination. The model exhibited excellent discriminatory capability by accurately predicting the probability of CSS across these years, facilitated by the extremely precise predictive models of both sets.

The NRI values were observed at 0.313 (95% CI = 0.199–0.435), 0.370 (95% CI = 0.257–0.463), and 0.305 (95% CI = 0.216–0.383) for 3, 5, and 8 years of follow-up examinations, respectively, in the training cohort. In the validation cohort, the respective values were noted to be 0.169 (95% CI = 0.042–0.308), 0.249 (95% CI =  0.087–0.388), and 0.257 (95% CI = 0.047–0.401). These values were indicative of the substantial improvement brought about by the nomogram in terms of prognosis prediction. Likewise, the IDI values across 3, 5, and 8 years of follow-up were 0.033, 0.042, and 0.049 in the training cohort (*p* < 0.01) and 0.021, 0.034, and 0.041 in the validation cohort (*p* < 0.01), respectively. The acquired data indicated that the new model exhibited a superior predictive performance.

**Figure 4:**
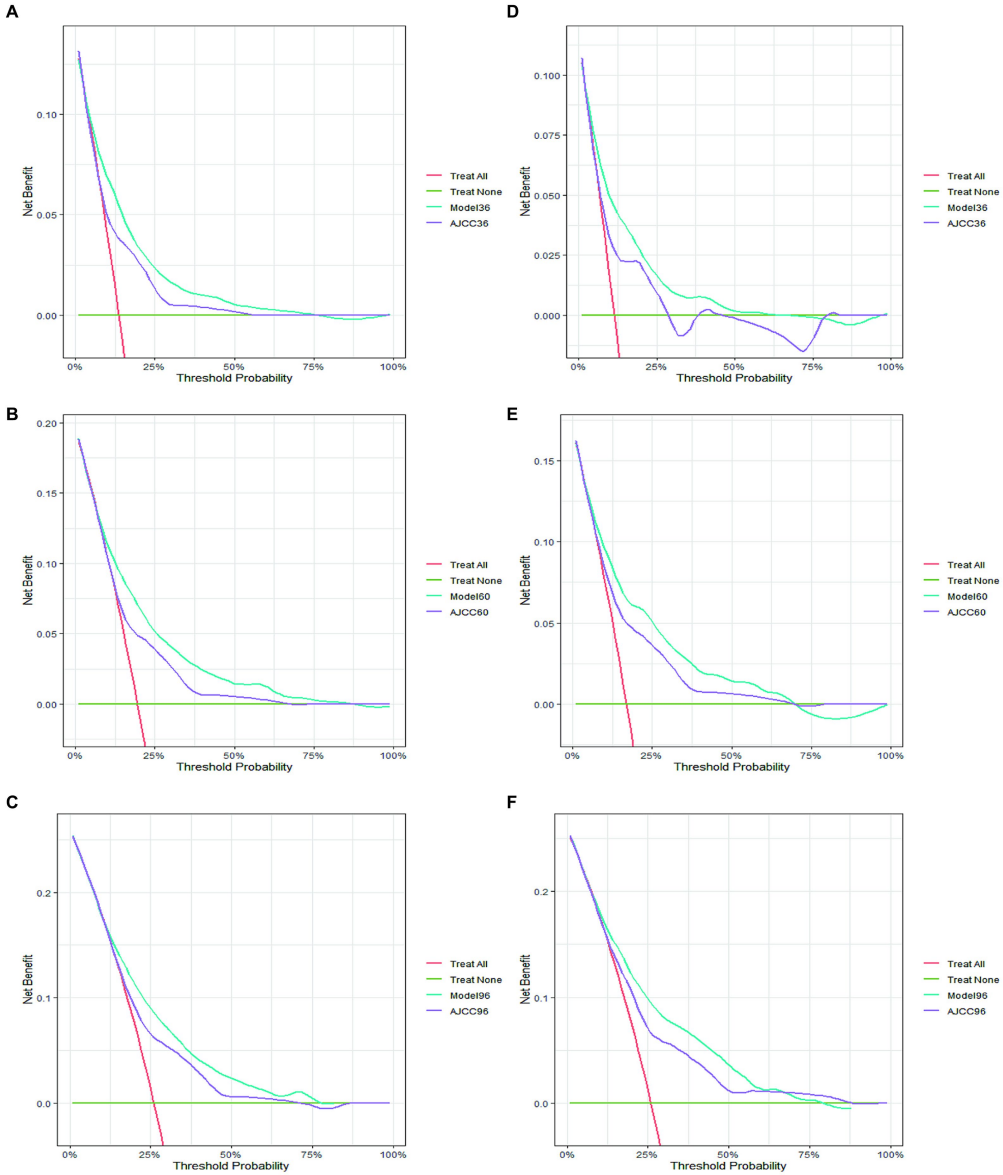
The decision curve analysis of 3-year **(A)**, 5-year **(B)**, and 8-year **(C)** CSS in the training cohort and 3-year **(D)**, 5-year **(E)**, and 8-year **(F)** CSS in the validation cohort.

The DCA curves were generated for the post-diagnosis follow-up periods of 3, 5, and 8 years in both cohorts, as depicted in Figure 4. The established nomogram exhibited a superior net benefit in the prognosis assessment of CM patients in comparison to the AJCC system, which underscores its pronounced clinical utility in guiding prognostic assessments.

## Discussion

In the course of this study, a comprehensive nomogram was developed, incorporating clinicopathological parameters with the AJCC staging system. This nomogram serves the purpose of accurately assessing the definitive CSS probabilities across 3, 5, and 8 years in individuals with CM. Seven parameters including age, race, AJCC stage, histologic types, radiotherapy, surgery, and chemotherapy exhibited a strong correlation with the CSS of adults with CM, establishing them as independent prognostic factors. Furthermore, analyses such as the ROC curve, DCA, and calibration plot underscored the considerable predictive potential of the nomogram. The acquired data was indicative of the value of the nomogram as a tool used by physicians to determine the most suitable treatment approach for their patients.

This model highlighted the substantial effect of the AJCC staging on the prognostic score, indicated by the overall favorable prognoses associated with most individuals with an early-stage (I and II) CM. Additionally, the prognoses of stages III and IV CM were observed to be poor. This observation aligns with the findings of a prior study by Shields et al. (7). Several retrospective studies on large patient populations have consistently reported that the characteristics used for AJCC staging also serve as prognostic indicators for the likelihood of developing distant metastasis (7, 20, 21). Therefore, early diagnosis, especially when the tumor is small, emerges as an important and potentially lifesaving measure.

Furthermore, it was found that race exerted a strong influence on the survival outcome of adult patients with CM. Research indicates that CM is more common in Caucasian people compared to other races, being Caucasian has also been identified as a poor prognostic factor for CM in prior studies (2, 22). Hu et al. (23) evaluated that the decreased incidence of melanoma in non-Caucasian people could be linked to the protective influence of skin and eye pigmentation, as black individuals have a considerably lower risk of CM than Caucasian people. In the multiracial US environment, cancer survival rates vary immensely among different races, and this variance is even more evident between Caucasian people and Black people.

The acquired data indicated that the histologic type of the CM could also function as an important prognostic determinant in the clinical workup. Additionally, patients with spindle cell type melanoma were more likely to have a better prognosis for survival than those with either mixed or epithelioid type melanoma, with the latter having a worse prognosis. These findings are congruent with those of prior studies (10, 24, 25). The likely explanation lies in the biology of different cell types. Spindle cell-melanomas are more regular, with a spindle shape, relatively slow growth rate, and less aggressive. This type of tumor cell is generally less susceptible to metastasis. Epithelial-cell melanomas have epithelioid-shaped tumor cells that are closely packed and sometimes form a nest or layered structure. They proliferate fast, have obvious atypia, and are prone to metastasize.

The data acquired in this research indicated a higher morbidity rate associated with CM in males than in females, which is in accordance with the outcomes of prior research from Canada, Australia, and Germany (3, 26, 27). However, the relationship between sex and prognosis of patients with CM was not statistically significant in our study. This, as several studies have reported, could be ascribed to varied genetic predispositions in sex, including the hormonal profile of women, and the greater exposure chances of males to chemical and solar carcinogens (28, 29). Interestingly, Damato and Coupland (30) have shown that the presentation of CM tends to be larger and more posterior in males than in females. However, further comparison of the characteristics of CM based on sex is essential to adequately address these questions.

The nomogram illustrated that age was correlated with the survival of patients with CM. This correlation was evident by the presence of higher CSS among patients aged 18–50 years compared to other age groups. Prior research has suggested that older age groups exhibit higher incidences of CM along with adverse prognoses (11, 27). The poorer survival in older individuals was ascribed to the aging microenvironment that may have a significant impact on tumor progression. On one hand, normal aging-associated changes in immune and stromal populations may play a crucial function in promoting the transition of tumor cells from an initial or slow-growing state to an increasingly invasive and metastatic one (31). On the other hand, considering the specificity of age-related health conditions, there are numerous side effects linked to therapies that can often pose life-threatening risks in elderly individuals.

However, despite the growing understanding of CM biology, the therapeutic strategies remain controversial, and existing treatments have not remarkably improved patient survival. Our findings indicate that only radiotherapy can markedly enhance the survival rate of adult patients with CM, while surgery and chemotherapy are associated with a heightened risk of adverse prognosis. It is worth noting that historically, enucleation stood as the sole available treatment for a considerable period. Conservative treatments have gradually started replacing enucleation in the last few decades. Radiotherapy has become the first-line treatment for small to medium-sized melanomas with a local control of approximately 95%. Various forms of radiotherapy are safe and effective for those with localized disease and can preserve the affected eye (32–34). Multiple studies have demonstrated that radiotherapy, considered the most crucial therapy for CM, can extend the survival time of patients (35). Surprisingly, our study found that surgery was an adverse risk factor. Jang et al. (35) and Liu et al. (9) reported that patients treated with surgery showed an overall worse survival rate than those treated with radiotherapy. One possible explanation is that surgery was performed only in cases with large CM, with CM surrounding the optic nerve, or suspected extraocular extension (36). Such cases usually have worse outcomes than small-and medium-sized CM (7, 37). Furthermore, intraoperative manipulation may have accelerated the micrometastasis of the tumor by the pulling and squeezing of the vascular tissue (38). However, in comparison to these findings, Shields and Shields (39) reported that surgery may result in favorable survival outcomes in medium-sized CM. In addition, due to the relatively insufficient sample size of surgery we included, the accuracy of the results may be affected. Thus, the prognostic impact of surgery on patients with CM remains rather inconclusive and should be explored further. Moreover, in our study, it was observed that the prognosis of individuals who underwent chemotherapy was worse. Chemotherapy has now been applied clinically to treat distant metastatic CM, especially for hepatic metastatic cancers, which have a poorer prognosis and limited therapeutic options with low response rates (1). Fane and Weeraratna (31) pointed out that chemotherapy could offer initial benefits in numerous cases, but it could subsequently contribute to accelerated immunosenescence and increased residual disease in patients. In recent years, the emphasis of CM treatment is increasingly shifting towards complex and personalized therapies, particularly in metastatic scenarios. Novel therapeutic strategies, including molecular-targeted therapy and immunotherapy, present promising avenues for enhancing the survival rates of patients with metastatic CM (6, 40).

This study is limited in certain respects. Firstly, this was a retrospective study potentially prone to selection and information biases. The exclusion of data with missing or unclear information further exacerbates the risk of selection bias. Secondly, due to the absence of gene expression profile and chromosomal factors of primary uveal melanomas in the SEER database (i.e., BAP1 germline mutations, monosomy 3 and gain of chromosome 8q), which are key factors for clinical prognosis in uveal melanoma (41, 42), the effectiveness of the nomogram may be impaired. Several important prognostic factors, including relapse free survival, microvascular density and mitotic index were not taken into consideration in the study (42). Additionally, detailed information about the therapy, such as the surgical approach and the dose of radiotherapy was unclear. Finally, the rarity of CM results in a shortage of external data from different regions, necessitating further validation with external data to confirm the generalizability of the results. Future prospective studies should be conducted to address these limitations by testing the nomogram.

## Conclusion

In conclusion, utilizing the clinical risk factors screened in an extensive population-based cohort, the first practical nomogram for CM was established. It was capable of objectively and accurately predicting the individualized risk of CM. The nomogram not only exhibited ample discriminatory and calibration capacity but also considerable clinical effectiveness. It exhibited the capability to function as a user-friendly tool for clinicians, facilitating personalized postoperative prognostic assessment and aiding in the identification of treatment approaches for adult patients with CM.

## Data availability statement

The raw data supporting the conclusions of this article will be made available by the authors, without undue reservation.

## Ethics statement

The studies involving humans were approved by The First Affiliated Hospital of Jinan University. The studies were conducted in accordance with the local legislation and institutional requirements. Written informed consent for participation was not required from the participants or the participants’ legal guardians/next of kin because patient information in the SEER program is de-identified and publicly available.

## Author contributions

XC: Writing – original draft. JZ: Writing – original draft. YO: Data curation, Writing – original draft. JC: Funding acquisition, Writing – original draft. JL: Data curation, Methodology, Project administration, Resources, Supervision, Writing – review & editing. QZ: Formal analysis, Funding acquisition, Supervision, Writing – review & editing.
